# Effect of preconceptional DHEA treatment on in vitro fertilization outcome in poor ovarian respond women: study protocol for a randomized controlled trial

**DOI:** 10.1186/s13063-018-3146-x

**Published:** 2019-01-15

**Authors:** Wei Wang, Hong Liu, Jing Li, Daimin Wei, Jiangtao Zhang, Jianfeng Wang, Jinlong Ma, Yuhua Shi, Zi-Jiang Chen

**Affiliations:** 1 Center for Reproductive Medicine, Key Laboratory of Reproductive Endocrinology, Ministry of Education, and National Research Center for Assisted Reproductive Technology and Reproductive Genetics, Shandong Provincial Clinical medicine Research Center for reproductive health, Shandong Provincial Hospital Affiliated with Shandong University, Shandong University, No. 157 Jing Liu Street, Shizhong district, Jinan, 250012 China; 2 Center for Reproductive Medicine, The Second Clinical Medical College, Yangtze University, No. 2 People Street, Jingzhou district, Jingzhou, 434020 China; 3 Center for Reproductive Medicine, School of Medicine, Shanghai Key Laboratory for Assisted Reproduction and Reproductive Genetics, Shandong University, Shanghai Jiao Tong University, No. 845 Lingshan Road, Pudong new district, Shanghai, 310101 China

**Keywords:** Dehydroepiandrosterone, In vitro fertilization, Poor ovarian response, Live birth

## Abstract

**Background:**

Women undergoing in vitro fertilization (IVF) or intracytoplasmic sperm injection (ICSI) with poor ovarian respond (POR) always have very low clinical pregnancy rates. In previous data, dehydroepiandrosterone (DHEA) was suggested as a promising treatment and maybe has a good pregnancy outcome. But there is no sufficient evidence from randomized clinical trials evaluating the effect of DHEA preconceptional treatment on live birth in POR.

**Methods:**

This trial is a multicenter active-placebo double-blind clinical trial (1:1 treatment ratio of active versus placebo). The infertile POR patients undergoing IVF or ICSI will be enrolled and randomly assigned to two parallel groups. Participants in these two groups will be given 4–12 weeks’ treatment of DHEA or placebo, respectively. The primary outcome is live birth rate.

**Discussion:**

The results of this study will provide evidence for the effect of preconceptional DHEA treatment on IVF outcome in POR.

**Trial registration:**

Chinese Clinical Trial Registry, ChiCTR-IPR-15006909. Registered on November 9, 2015.

**Electronic supplementary material:**

The online version of this article (10.1186/s13063-018-3146-x) contains supplementary material, which is available to authorized users.

## Background

The management of poor ovarian respond (POR) remains a serious dilemma for fertility clinicians. The incidence of POR, which was associated with very low clinical pregnancy rate (CPR), was reported to be 9–24% [1]. Among infertile women in vitro fertilization (IVF) cycles. CPR was only 14% and the cycle cancellation rate of no available embryos was as high as 40% for women with fewer than five oocytes [2]. It was confirmed that the outcomes of IVF/intracytoplasmic sperm injection (IVF/ICSI) would be improved with increasing number of oocytes retrieved, especially for those with poor ovarian response or advanced maternal age [3].

Various approaches have been used to obtain more mature oocytes and available embryos for POR. Different stimulation protocols [4–7], increasing gonadotropin dosage [8, 9], combining pretreatment protocol [10–12], and usage of adjuvant [13, 14] have been tried in IVF/ICSI cycles. But there is no evidence from randomized clinical trials evaluating the effect of dehydroepiandrosterone (DHEA) preconceptional treatment on live birth in POR.

Androgen in the ovarian microenvironment plays an important role in early follicular development and granulosa cell proliferation. Administration of androgen was suggested as a promising treatment. Androgens can upregulate the follicular follicle-stimulating hormone (FSH) receptor expression, augment follicular sensitivity to FSH stimulation, and increase the number of antral follicles and mature oocytes [15]. Clinical observation [16] indicates that the lack of androgen is associated with low ovarian response and pregnancy rate during IVF.

The most widely used androgen in the clinic is DHEA. DHEA has low androgen activity but can transform highly active androgen *in vivo*; meanwhile, the incidence of adverse reactions is low. Numerous reports indicated that DHEA supplementation in patients with decreased ovarian reserve or POR was helpful to improve ovarian reserve parameters [17, 18], augment ovarian response [19], reduce age-related aneuploidy [20], and increase pregnancy rate [21]. However, most of the studies were based on retrospective or observational data or both. Several randomized controlled trials (RCTs) were criticized for inappropriate study design, the varied definition of POR, and duration of DHEA [22]. There is an urgent need for large-scale, well-designed confirmatory studies to prove the efficacy of DHEA before it could be recommended for routine use.

The purpose of this multicenter randomized placebo-controlled double-blind clinical trial is to evaluate the effect of DHEA treatment for 12 weeks before IVF/ICSI on the live birth rate in infertile POR.

## Methods/Design

### Study design

**Fig. 1:**
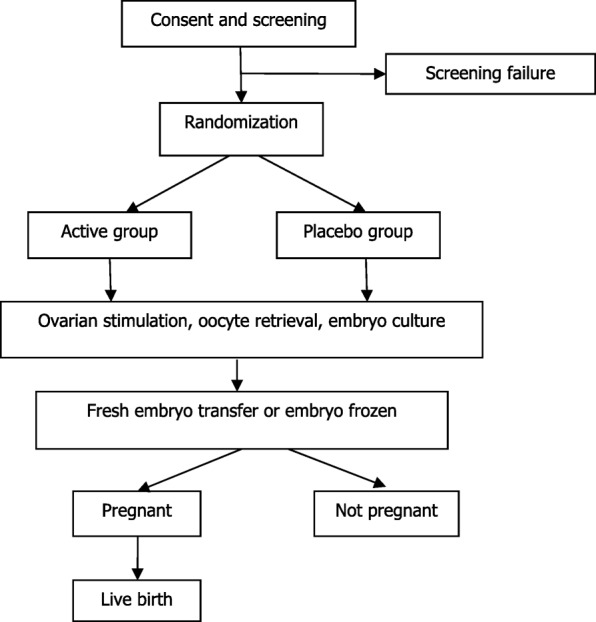
Study flowchart

This study has a multicenter, randomized active-placebo, double-blind design. Eligible patients will be randomly assigned to active group or placebo group with a 1:1 ratio. Figure 1 shows a flowchart of the study design.

### Study sites

This clinical trial involves eight hospitals in China. Live birth rate of active group versus placebo group will be compared in a pool of 710 infertile POR patients undergoing their sequence cycle of IVF/ICSI. The study will be conducted in accordance with Good Clinical Practice guidelines and the Declaration of Helsinki. The study has been approved by the ethics committees at all hospitals and facilities.

**Fig. 2:**
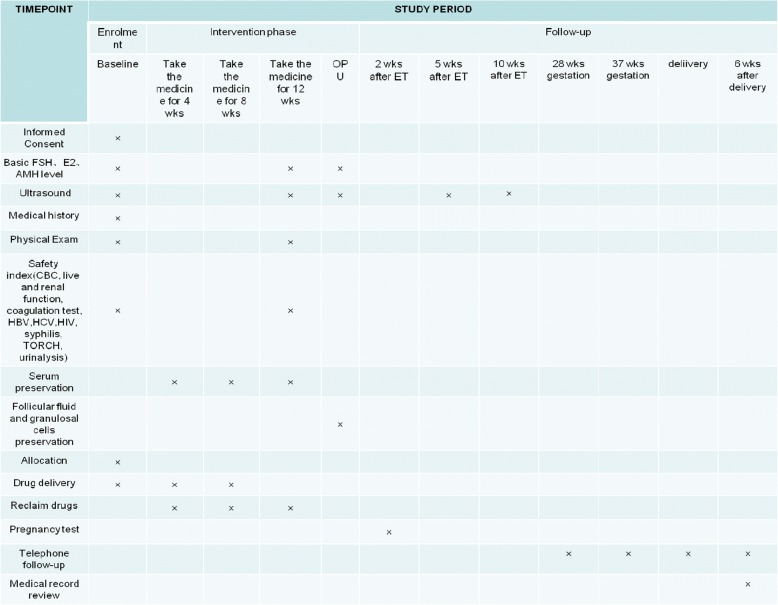
Standard Protocol Items: Recommendations for Interventional Trials (SPIRIT) figure

The trial and study plan will be declared to all participants at their first visit. If patients are eligible and interested in participating, the couples will be asked to sign an informed consent form giving permission to use their results anonymously in future studies. Reporting of the study results will follow the 2010 revised CONSORT (Consolidated Standards of Reporting Trials) statement [23]. An overview of specific measurements and time points of data collection can be found in the SPIRIT (Standard Protocol Items: Recommendations for Interventional Trials) Figure (Fig. 2, Additional file 1).

### Training of investigators

All investigators are required to undertake mandatory training in the protocol, Good Clinical Practice, and the use of CONSORT if they had no recent certifications.

#### Study population

The study will include patients with POR. Inclusion criteria are (1) women who had tried to become pregnant for at least 1 year, (2) women who will undergo IVF or ICSI, and (3) women with POR diagnosed according to the Bologna consensus [24].

Exclusion criteria are (1) women who had failed in three or more IVF cycles with ovarian stimulation and oocyte pickup, (2) women with diagnosed uterine malformation or abnormality, (3) women with diagnosed hydrosalpinx in hysterosalpingography, (4) women who had a history of two or more spontaneous miscarriages (biochemical miscarriage does not count in), (5) couples with female or male abnormal karyotype, (6) women who had undergone chemotherapy with cytotoxic agents, (7) women who had undergone pelvic radiotherapy, (8) women who had taken DHEA before enrollment, (9) women with a history of epilepsy, and (10) women who are allergic to DHEA.

### Determination of the sample size

The sample size calculation is based on the live birth rate. Data from our hospital indicated that the live birth rate of infertile POR undergoing IVF/ICSI cycles was 20%. In our present study, we plan to test the primary hypothesis of a difference of 10 in the live birth rate for the two randomization arms. A sample size of 294 prospectively enrolled participants in each randomization arm will yield a statistical power of 80 at a *P* value of less than 0.05 to demonstrate a significant difference in live birth rates of 10 between treatment arms. In consideration of a dropout rate of 20%, we will enroll 355 participants each group.

### Randomization and blinding

Eligible participants will be randomly assigned to one of the two study arms at a ratio of 1:1 according to an Excel table randomization list generated by a data coordinative center (DCC) staff who are independent of the study group, and the allocation will be input into the online database central grouping system. The random number list is kept strictly confidential by the DCC staff, and the researchers who are in charge of the enrollment have no access to the list.

Both the placebo and active component are manufactured by the pharmacist who was not involved in the subjects’ clinical management. Except for the active ingredients, the rest of the excipient and the appearance and odor are the same. The pharmacist packaged the DHEA and identical placebo capsules according to the randomization list and labeled the drug packs with subject numbers only. After screening the patients eligible for the entry, the researcher will log into the database to obtain the patient’s research number. Then the pharmacist will dispense the corresponding drug. The researchers and study participants will be blinded to the allocation until the end of the study.

#### Discontinuation criteria

Reasons for discontinuation of treatment may include, but are not limited to, the following: (1) participants become pregnant during the trial; (2) participants who have experienced some complications or serious side effects; (3) participants who do not comply with the DHEA were defined by using less than 80% or more than 120% of the prescribed amount; using prohibited drugs as described in the protocol, such as growth hormone and glucocorticoids; (4) participants who request to withdraw from the trial.

#### Intervention

The study will be conducted in following stages. After a recruitment period prior to baseline assessment and randomization, DHEA/placebo will be given to patients three times a day orally from the first day of their menstrual period for 4–12 weeks. After that, participants will undergo their IVF/ICSI cycle. Finally, a live birth rate follow-up will be performed.

### Controlled ovarian hyperstimulation

A short protocol will be suggested to all patients. On cycle day 2, gonadotropin-releasing hormone (GnRH) agonist (Triptorelin; Tiantai Mountain Pharmaceutical Company, Chengdu, China) 0.05~0.1 mg will be initiated. From cycle day 4, all patients will be stimulated with gonadotrophin (Gn) (FSH/human menopausal gonadotrophin; Livon, Zhuhai, China) 150 or 225 IU/day. Both GnRH agonist and Gn continue until the day of human chorionic gonadotropin (hCG) administration. On stimulation day 6, transvaginal ultrasound scans and serum hormone tests will be performed. The dose of Gn will be adjusted according to follicular development and endocrinometry. Afterward, such monitoring will be performed every other day or every day. When more than two lead follicles reach at least 18 mm, 6000–10,000 IU of hCG will be administered to trigger final follicular maturation.

### Oocyte retrieval, in vitro fertilization, and embryo culture

Transvaginal ultrasound-guided ovum pickup takes place about 34–36 h after hCG administration. Oocytes will be inseminated by IVF or ICSI according to the quality of sperm. Embryo transfer (ET) is routinely performed on days 3 after fertilization at the cleavage stage, and a maximum of two top quality embryos will be transferred through a catheter by using transabdominal ultrasound guidance (full bladder). If the quality of the embryo is poor, ET will be performed on day 2 or 5. Blastocyst culture and vitrification will be performed for the surplus embryos. All of the operations will be performed by experienced clinicians.

### Luteal phase support

Dydrogesterone tablet (Duphaston, Abbott, Hoofddorp, The Netherlands) 20 mg twice daily orally or vaginal progesterone gel (Crinone gel, 8%, Merck Serono, Geneva, Switzerland) 90 mg once daily will be used for luteal phase support (LPS) from the day after oocyte retrieval for 2 weeks. After a positive pregnancy test (serum β-hCG ≥10 IU/L), LPS will be continued until at least 10 weeks’ gestation. If β-hCG is negative or if the pregnancy fails, LPS will be stopped. If a biochemical pregnancy has been achieved, ultrasound scan will be performed at 5–6 weeks after ET to assess clinical pregnancy and repeated at 12 weeks’ gestation to confirm ongoing pregnancy.

#### Outcome measurement

##### Primary outcome

The primary endpoint of study is live birth, defined as delivery of any neonate at at least 28 weeks’ gestation with signs of life after ET.

##### Secondary outcomes

Secondary outcomes will include (1) change of ovarian reserve index (basal serum FSH, anti-Müllerian hormone (AMH), and antral follicle counts), (2) indexes of ovarian response (total gonadotropin dosage, estradiol (E2) on the HCG trigger day, and retrieved oocyte number), (3) indicators of oocytes quality (mature oocyte rate, normal fertilization rate, and number of top-quality embryos), and (4) CPR and miscarriage rate.

Biochemical pregnancy is defined as a serum β-hCG level of at least 10 IU/L 2 weeks after ET. Clinical pregnancy is defined as detection of intrauterine gestation sac after 3 weeks after a biochemical pregnancy is confirmed. Ongoing pregnancy is defined as detection of a viable fetus with fetal heartbeat at 11–12 weeks’ gestation. Cumulative live birth will be defined as each woman having only one allowable live birth from the single IVF cycle.

##### Follow-up protocol

First pregnancy follow-up time point is at 12 weeks’ gestation when the participants come for their clinic visits. The presence of first-trimester pregnancy complications (Ovarian hyperstimulation syndrome (OHSS), miscarriage, ectopic pregnancy, or gestational trophoblastic disease or a combination of these) will be evaluated by inspecting medical records and be recorded by completing the case report form. Moderate OHSS is diagnosed when ultrasonographic ascites is present in addition to abdominal distension and discomfort with or without nausea, vomiting, or diarrhea or a combination of these. Severe OHSS is diagnosed when there is clinical evidence of ascites and/or hydrothorax or breathing difficulties with or without hemoconcentration, coagulation abnormalities, and diminished renal function.

Second pregnancy follow-up time point is at 28 weeks’ gestation. The second-trimester pregnancy complications (prenatal diagnosis test results, abortion, gestational diabetes, preeclampsia/eclampsia, incompetent cervix, premature rupture of membrane, or placenta abruption or a combination of these) will be followed up by telephone call.

Third pregnancy follow-up time point is at 37 weeks’ gestation. The third-trimester pregnancy complications (preterm labor, placenta abruptio, placenta accreta, placenta previa, preeclampsia/eclampisa, intrauterine growth retardation, premature rupture of membrane, or abnormality of amniotic fluid or a combination of these) will be followed up by telephone call.

Fourth follow-up time point is at delivery. Participants will be required to inform investigators of the delivery time. The obstetrical and perinatal complications (gestational age, delivery mode, placenta abnormality, or delivery complications or a combination of these) as well as neonatal information (gender, birth weight, and birth defect) will be obtained and recorded to complete designed forms. If possible, copies of the obstetric medical records will be performed for the study chart source documents.

The fifth and final follow-up time point is at 6 weeks after delivery. Postpartum complications (postpartum depression, infection, and late postpartum hemorrhage) and neonatal complications (neonatal respiratory distress syndrome, neonatal jaundice, neonatal infection, neonatal death, and neonatal hospitalization) are followed up by telephone call.

##### Adverse events

Adverse events are defined as any untoward or unfavorable medical occurrences associated with the subject’s participation during the research regardless of whether they are considered related to the study intervention. Serious adverse events are defined as events that are temporally associated with the subject’s participation in research: death, life-threatening or severely or permanently disabling events, events requiring in-patient hospitalization or prolongation of existing hospitalization, pregnancy loss after 20 weeks’ gestation, neonatal death up to 6 weeks after delivery, congenital anomaly or birth defect, or any events deemed serious by the local principal investigator.

All of the adverse events will be recorded in detail. Serious adverse events will be reported to the principal investigator immediately, and appropriate measures will be initiated instantly. The ethics committee will determine whether the adverse event is likely to have been associated with the experimental drug and whether it is necessary to break blinding codes.

### Data management

All data will be collected with a standard case report form developed in the web-based data entry system by the double-entry method. Before being input into the database, data will be de-identified. Paper files will be kept in a locked filing cabinet in the treating hospital. Electronic documents will be stored in a password-protected computer, and access will be restricted to the principal investigator. The study site monitor of Shandong University will check the authenticity, accuracy, and integrity of the data regularly to ensure the accuracy of collected data. The data will be stored for at least 5 years after publication.

All data will be supervised by independent statisticians from Shandong University. The clinical research must be carried out under the rules on China’s New Drug Examination and Approval and Management Standard of the Clinical Test of Drugs (Good Clinical Practice). Data monitors will check and review the quality of data collected in the research accordingly.

#### Data analysis plan

Statistical analysis will be performed by using Statistical Package for the Social Sciences Version 22.0 (SPSS Inc., Chicago, IL, USA). Normally distributed continuous variables are expressed as mean ± standard deviation with a Wilcoxon rank-sum test for testing between-group differences. Non-normally distributed continuous variables are expressed as medians and ranges, and categorical data are described as frequency, percentage, and constituent ratio.

The primary analysis will use an intention-to-treat analysis approach to examine differences in the live birth rate between the two treatment arms by the Pearson chi-squared test. Secondary efficacy parameters and safety parameters (pregnancy rate, OHSS rate, and so on) will be analyzed by using the Pearson chi-squared test. The Wilcoxon rank-sum test will be performed to compare continuous parameters (retrieved oocyte number, gonadotropin dosage, E2 level on the hCG trigger day, and so on). The analysis of covariance (ANCOVA) tests will be used to compare the indicators (basic FSH, E2 level, and AMH level) pre- and post-treatment between-group, and pre-post paired *t* tests will be used within-group.

A secondary per-protocol analysis will be performed according to the actual treatment that subjects received.

Results will be presented as point estimates and 95% confidence intervals; the level of significance will be set at 5%.

The flowchart of this study is presented in Fig. 1, and the SPIRIT checklist is included as Fig. 2.

## Discussion

This study will compare the efficacy of 12-week DHEA treatment prior to IVF in POR. We plan to enroll 710 subjects from eight academic IVF centers in the Shandong Province. The result of this large multicenter randomized trial will provide level I evidence for the strategy of DHEA treatment for 12 weeks before IVF in poor ovarian responders.

DHEA treatment before IVF in POR has attracted increasing attention and has been considered promising for POR [25]. However, there are still great gaps in the literature to illuminate the risk/benefit ratio of this strategy, which includes multiple steps of treatment. Some research [20, 26–29] showed that DHEA supplementation improved the indicate of ovarian reserve, augmented ovarian response, and increased the clinical pregnancy rate. Meanwhile, the others [30–32] showed there were no such benefits. Some small-scale RCTs [28, 30, 31, 33] have been conducted to compare the efficacy of DHEA treatment before IVF. However, the varied definition of the patient population and primary outcome may result in the heterogeneity of comparisons. Additionally, the lack of rigorous design strategies, large scale, and high quality also make it difficult to reach any consistent conclusion [34].

This study is a multicenter randomized placebo-controlled double-blind clinical trial. In this research, we define poor ovarian responder according to the Bologna consensus [24], which is widely applied in clinical research after the recommendation. In 2003, the European Society of Human Reproduction and Embryology (ESHRE) recommended the singleton live birth rate as the evaluation index of assisted reproductive technology (ART) or non-ART reproductive outcome [35]. A healthy baby is the purpose of couples. That is the reason we chose live birth rate as the primary endpoint for this study.

Despite the lack of convincing evidence, DHEA supplementation is being used by more and more IVF centers around the world for POR. Large-scale, randomized placebo-controlled, blind, clinical trials are urgent to suggest physicians whether or not to use DHEA and how to use it. This study is expected to provide a reliable answer.

## Trial status

The study was conceived and designed in 2015. Enrollment began in April 2016 and was expected to end in December 2018. At the time of manuscript preparation, more than 500 subjects had been enrolled. Enrollment in this study was ongoing at the time of manuscript submission.

## Additional file

Additional file 1: SPIRIT (Standard Protocol Items: Recommendations for Interventional Trials) checklists. (DOCX 41 kb)

## Abbreviations

AMH: Anti-Müllerian hormone
ART: Assisted reproductive technology
CONSORT: Consolidated Standards of Reporting Trials
CPR: Clinical pregnancy rate
DCC: Data coordinative center
DHEA: Dehydroepiandrosterone
E2: Estradiol
ET: Embryo transfer
FSH: Follicle-stimulating hormone
Gn: Gonadotropin
GnRH: Gonadotropin-releasing hormone
hCG: Human chorionic gonadotropin
ICSI: Intracytoplasmic sperm injection
IVF: In vitro fertilization
LPS: Luteal phase support
OHSS: Ovarian hyperstimulation syndrome
POR: Poor ovarian respond
RCT: Randomized controlled trial
SPIRIT: Standard Protocol Items: Recommendations for Interventional Trials

## References

[1] Keay SD, Liversedge NH, Mathur RS, Jenkins JM (1997). Assisted conception following poor ovarian response to gonadotrophin stimulation. Br J Obstet Gynaecol..

[2] Saldeen P, Kallen K, Sundstrom P (2007). The probability of successful IVF outcome after poor ovarian response. Acta Obstet Gynecol Scand..

[3] Briggs R, Kovacs G, MacLachlan V, Motteram C, Baker HW (2015). Can you ever collect too many oocytes?. Hum Reprod..

[4] Tazegul A, Gorkemli H, Ozdemir S, Aktan TM (2008). Comparison of multiple dose GnRH antagonist and minidose long agonist protocols in poor responders undergoing in vitro fertilization: a randomized controlled trial. Arch Gynecol Obstet..

[5] Pu D, Wu J, Liu J (2011). Comparisons of GnRH antagonist versus GnRH agonist protocol in poor ovarian responders undergoing IVF. Hum Reprod..

[6] Kuang Y, Chen Q, Hong Q, Lyu Q, Ai A, Fu Y (2014). Double stimulations during the follicular and luteal phases of poor responders in IVF/ICSI programmes (Shanghai protocol). Reprod BioMed Online.

[7] Mochtar MH, Danhof NA, Ayeleke RO, Van der Veen F, van Wely M (2017). Recombinant luteinizing hormone (rLH) and recombinant follicle stimulating hormone (rFSH) for ovarian stimulation in IVF/ICSI cycles. Cochrane Database Syst Rev..

[8] Garcia-Velasco JA, Isaza V, Requena A, Martinez-Salazar FJ, Landazabal A, Remohi J (2000). High doses of gonadotrophins combined with stop versus non-stop protocol of GnRH analogue administration in low responder IVF patients: a prospective, randomized, controlled trial. Hum Reprod.

[9] Lefebvre J, Antaki R, Kadoch IJ, Dean NL, Sylvestre C, Bissonnette F (2015). 450 IU versus 600 IU gonadotropin for controlled ovarian stimulation in poor responders: a randomized controlled trial. Fertil Steril..

[10] Cedrin-Durnerin I, Guivarc’h-Leveque A, Hugues JN (2012). Groupe d’Etude en Medecine et Endocrinologie de la R. Pretreatment with estrogen does not affect IVF-ICSI cycle outcome compared with no pretreatment in GnRH antagonist protocol: a prospective randomized trial. Fertil Steril..

[11] Sobotka V, Streda R, Mardesic T, Tosner J, Heracek J (2014). Steroids pretreatment in assisted reproduction cycles. J Steroid Biochem Mol Biol..

[12] Smulders B, van Oirschot SM, Farquhar C, Rombauts L, Kremer JA. Oral contraceptive pill, progestogen or estrogen pre-treatment for ovarian stimulation protocols for women undergoing assisted reproductive techniques. Cochrane Database Syst Rev. 2010:CD006109. 10.1002/14651858.CD006109.pub2 20091585

[13] Nagels HE, Rishworth JR, Siristatidis CS, Kroon B. Androgens (dehydroepiandrosterone or testosterone) for women undergoing assisted reproduction. Cochrane Database Syst Rev. 2015:CD009749. 10.1002/14651858.CD009749.pub2 PMC10559340 26608695

[14] Duffy JM, Ahmad G, Mohiyiddeen L, Nardo LG, Watson A. Growth hormone for in vitro fertilization. Cochrane Database Syst Rev. 2010:CD000099. 10.1002/14651858.CD000099.pub3 PMC7058116 20091500

[15] Nielsen ME, Rasmussen IA, Kristensen SG, Christensen ST, Mollgard K, Wreford Andersen E (2011). In human granulosa cells from small antral follicles, androgen receptor mRNA and androgen levels in follicular fluid correlate with FSH receptor mRNA. Mol Hum Reprod..

[16] Sunkara SK, Coomarasamy A (2011). Androgen pretreatment in poor responders undergoing controlled ovarian stimulation and in vitro fertilization treatment. Fertil Steril..

[17] Yilmaz N, Uygur D, Inal H, Gorkem U, Cicek N, Mollamahmutoglu L (2013). Dehydroepiandrosterone supplementation improves predictive markers for diminished ovarian reserve: serum AMH, inhibin B and antral follicle count. Eur J Obstet Gynecol Reprod Biol..

[18] Zhang HH, Xu PY, Wu J, Zou WW, Xu XM, Cao XY (2014). Dehydroepiandrosterone improves follicular fluid bone morphogenetic protein-15 and accumulated embryo score of infertility patients with diminished ovarian reserve undergoing in vitro fertilization: a randomized controlled trial. J Ovarian Res..

[19] Casson PR, Lindsay MS, Pisarska MD, Carson SA, Buster JE (2000). Dehydroepiandrosterone supplementation augments ovarian stimulation in poor responders: a case series. Hum Reprod..

[20] Gleicher N, Weghofer A, Barad DH (2010). Dehydroepiandrosterone (DHEA) reduces embryo aneuploidy: direct evidence from preimplantation genetic screening (PGS). Reprod Biol Endocrinol..

[21] Barad D, Brill H, Gleicher N (2007). Update on the use of dehydroepiandrosterone supplementation among women with diminished ovarian function. J Assist Reprod Genet..

[22] Fouany MR, Sharara FI (2013). Is there a role for DHEA supplementation in women with diminished ovarian reserve?. J Assist Reprod Genet..

[23] Moher D, Hopewell S, Schulz KF, Montori V, Gotzsche PC, Devereaux PJ (2012). Consort. CONSORT 2010 explanation and elaboration: updated guidelines for reporting parallel group randomised trials. Int J Surg..

[24] Ferraretti AP, La Marca A, Fauser BC, Tarlatzis B, Nargund G, Gianaroli L (2011). Definition EwgoPOR. ESHRE consensus on the definition of ‘poor response’ to ovarian stimulation for in vitro fertilization: the Bologna criteria. Hum Reprod..

[25] Farquhar C, Marjoribanks J, Brown J, Fauser B, Lethaby A, Mourad S (2017). Management of ovarian stimulation for IVF: narrative review of evidence provided for World Health Organization guidance. Reprod BioMed Online.

[26] Xu B, Li Z, Yue J, Jin L, Li Y, Ai J (2014). Effect of dehydroepiandrosterone administration in patients with poor ovarian response according to the Bologna criteria. PLoS One.

[27] Gleicher N, Ryan E, Weghofer A, Blanco-Mejia S, Barad DH (2009). Miscarriage rates after dehydroepiandrosterone (DHEA) supplementation in women with diminished ovarian reserve: a case control study. Reprod Biol Endocrinol..

[28] Jirge PR, Chougule SM, Gavali VG, Bhomkar DA (2014). Impact of dehydroepiandrosterone on clinical outcome in poor responders: A pilot study in women undergoing in vitro fertilization, using bologna criteria. J Hum Reprod Sci..

[29] Tsui KH, Lin LT, Horng HC, Chang R, Huang BS, Cheng JT (2014). Gene expression of cumulus cells in women with poor ovarian response after dehydroepiandrosterone supplementation. Taiwan J Obstet Gynecol..

[30] Kara M, Aydin T, Aran T, Turktekin N, Ozdemir B (2014). Does dehydroepiandrosterone supplementation really affect IVF-ICSI outcome in women with poor ovarian reserve?. Eur J Obstet Gynecol Reprod Biol..

[31] Yeung TW, Chai J, Li RH, Lee VC, Ho PC, Ng EH (2014). A randomized, controlled, pilot trial on the effect of dehydroepiandrosterone on ovarian response markers, ovarian response, and in vitro fertilization outcomes in poor responders. Fertil Steril..

[32] Vlahos N, Papalouka M, Triantafyllidou O, Vlachos A, Vakas P, Grimbizis G (2015). Dehydroepiandrosterone administration before IVF in poor responders: a prospective cohort study. Reprod BioMed Online.

[33] Wiser A, Gonen O, Ghetler Y, Shavit T, Berkovitz A, Shulman A (2010). Addition of dehydroepiandrosterone (DHEA) for poor-responder patients before and during IVF treatment improves the pregnancy rate: a randomized prospective study. Hum Reprod..

[34] Kolibianakis EM, Venetis CA, Tarlatzis BC (2011). DHEA administration in poor responders. Hum Reprod..

[35] Land JA, Evers JL (2003). Risks and complications in assisted reproduction techniques: Report of an ESHRE consensus meeting. Hum Reprod..

